# Contribution of neutrophil elastase to the lysis of obliterative thrombi in the context of their platelet and fibrin content

**DOI:** 10.1016/j.thromres.2010.05.007

**Published:** 2010-08

**Authors:** Gyöngyi Rábai, Nóra Szilágyi, Péter Sótonyi, Ilona Kovalszky, László Szabó, Raymund Machovich, Krasimir Kolev

**Affiliations:** a Department of Medical Biochemistry, Semmelweis University, Budapest, Hungary; b Department of Vascular Surgery, Semmelweis University, Budapest, Hungary; c 1st Department of Pathology, Semmelweis University, Budapest, Hungary; d Department of Physiology and Neurobiology, Institute of Biology, Eötvös Loránd University of Sciences, Budapest, Hungary; e Institute of Nanochemistry and Catalysis, Chemical Research Center, Hungarian Academy of Sciences, Budapest, Hungary

**Keywords:** DAPI, 4’,6-diamidino-2-phenylindole, GPIIb/IIIa, integrin α_IIb_β_3_, NE, neutrophil elastase, NE-FDP, NE-specific fibrin degradation products, PBS, phosphate buffered saline, SEM, scanning electron microscope, TBS, Tris buffered saline, Fibrin, Fibrinolysis, Leukocytes, Neutrophil elastase, Platelet, Thrombus

## Abstract

Leukocytes invade newly formed thrombi through interactions with platelets and fibrin and later contribute to the removal of fibrin deposits mainly through the action of neutrophil elastase. The present study attempts to express in quantitative terms the impact of neutrophils on the lytic processes in obliterative thrombi based on the local presence of elastase-specific fibrin degradation products (NE-FDP) in relation to the leukocyte, platelet and fibrin content of thrombi. Immunofluorescent detection of fibrin, NE-FDP and platelet antigens was performed in sections of thrombi from 28 patients subjected to thrombectomy in combination with DNA-staining for identification of nucleated cells. The digitalized fluorescent microscopic images were decomposed according to the color channel of each thrombus constituent. The integrated intensity values for all thrombus constituents were statistically evaluated with correlation, hierarchical agglomerative clustering , Hotelling's T^2^ and F-statistics. Association between NE-FDP and leukocyte content of thrombi is evidenced by a significant Pearson correlation coefficient of 0.71 (p = 0.00002). Cluster analysis reveals two classes of thrombi according to NE-FDP, leukocyte and platelet content and also two according to NE-FDP, leukocyte and fibrin content. When NE-FDP, fibrin and platelet content is normalized to the leukocyte count in the same thrombus, clusters with platelet-related thrombolytic resistance (inversely related NE-FDP and platelet content) and advanced cell-dependent thrombolysis (inversely related NE-FDP and fibrin content) are identified. These distinct patterns of thrombus constituents are snapshots of characteristic stages in the cell-dependent thrombolysis, which indicate a clot-stabilizing role for platelets in this process similar to their impact on the plasmin-dependent lysis.

## Introduction

Thrombus growth at sites of vascular injury or over atherosclerotic plaques is initiated by the shear-dependent platelet aggregation involving the priming adhesive role of the platelet glycoprotein (GP) Ib receptor and subsequent stabilization of the plug through the integrin α_IIb_β_3_ (GP IIb/IIIa) activation by soluble agonists [1,2]. Adherent platelets recruit leukocytes from circulating blood, predominantly neutrophils, representing 76 % of the leukocytes in thrombi, and monocytes (16 %) [3] followed by activation of the thrombus-bound cells [4]. Upon activation monocytes express tissue factor and form tissue factor-rich microparticles, which together with the damaged tissue-derived tissue factor promote fibrin formation as a scaffold of the growing thrombus [5]. Thrombogenesis is supported by the platelet activating effects of cathepsin G released in the course of degranulation of activated neutrophils together with neutrophil elastase (NE) [6,7]. In contrast to the prothrombotic interplay of thrombus-bound cells, in later stages of thrombus formation neutrophil adhesion seems to restrict thrombus growth [8] through removal of the deposited fibrin, which can be attributed to phagocytotic activity [9,10] and/or extracellular proteolysis based on released proteases [11,12]. The major fibrinolytic enzymes of human leukocytes are known to be NE and cathepsin G [13,14], the catalytic efficiency of NE in fibrin dissolution being ten-fold higher than that of cathepsin G [15]. Because NE cleaves fibrin at peptide bonds different from the site of action of plasmin (the major classic fibrinolytic protease), NE-digested fibrin degradation products (FDP) can be distinguished immunologically from the plasmin-digested products. Using a monoclonal antibody raised against these NE-specific FDPs, elevation of the plasma levels of NE-digested FDPs has been observed in several inflammatory disease states [16], but little is known about the *in vivo* contribution of NE to the lytic process in thrombi. The present study was designed to express in quantitative terms the impact of neutrophils on the lytic processes in obliterative vascular thrombi based on the presence of NE-specific FDPs in the thrombus structure in relation to the leukocyte, platelet and fibrin content of thrombi.

## Materials and methods

### Patients

Twenty-eight patients (15 men and 13 women, mean age: 61 years; range: 46-88 with one extreme value of 19) subjected to thrombectomy were enrolled in the study. Twenty-five of them had obliterative thrombosis localized in large arteries (femoral, ileac, popliteal, brachial, radial, carotid) based on atherosclerosis in 22 cases and related to diabetes mellitus in 6 patients (in 11 cases the thrombus was in a previously implanted graft). Three patients had venous thrombosis (two of them with renal vein thrombus and one with pulmonary embolus). With the exception of the youngest patient with renal vein thrombosis, who had thrombophilia related to systemic lupus erythematosus, no other inherited or acquired thrombophilic state could be diagnosed in the examined group. At the time of thrombectomy all patients received heparin treatment, whereas in their history 12 patients were treated with low-molecular weight heparin, 17 patients with anti-aggregatory drugs and 7 patients with oral anticoagulant. In 2 cases clot lysis with tissue-type plasminogen activator was attempted before the thrombectomy. Written informed consent was obtained from all patients and the study protocol was approved by the institutional and regional ethical board.

### Scanning electron microscope (SEM) imaging of thrombi

Immediately after the surgery, 5 × 5 × 10 mm pieces of thrombi were placed into 10 ml 100 mM Na-cacodylate pH 7.2 buffer for 24 h at 4 °C. Following repeated washes with the same buffer thrombi were fixed in 1 v/v% glutaraldehyde for 16 h. The fixed thrombi were dehydrated in a series of ethanol dilutions (20 – 96 v/v%), 1:1 mixture of 96 v/v% ethanol/acetone and pure acetone followed by critical point drying with CO_2_ in E3000 Critical Point Drying Apparatus (Quorum Technologies, Newhaven, UK). The specimens were mounted on adhesive carbon discs, sputter coated with gold in SC7620 Sputter Coater (Quorum Technologies, Newhaven, UK) and images were taken with scanning electron microscope EVO40 (Carl Zeiss Gmbh, Jena, Germany).

### Immunohistochemistry

**Fig. 1:**
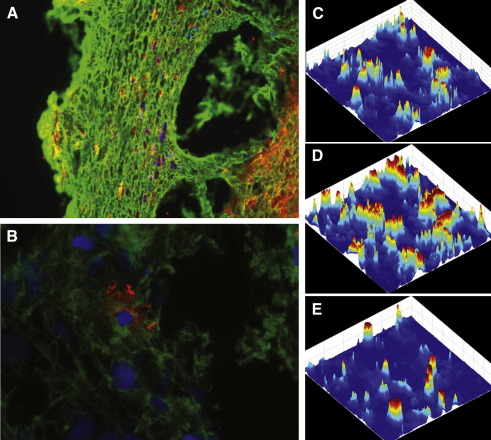
Fibrin, NE-digested fibrin and leukocyte content of arterial thrombi. Sections of surgically removed human thrombi were double-immunostained for fibrin (green) and NE-digested fibrin (red) as well as with a DNA-dye, DAPI (blue) as described in Methods. Images were taken at original magnification of × 20 (A) and × 60 (B). The pixel values of the three color channels (C: red, NE-FDP; D: green, fibrin; E: blue, nuclei) of an image taken as illustrated in panels A and B were decomposed according to the space coordinates of the thrombus section and their intensity is presented in a color-coded scale.

After surgery, the removed thrombus samples were frozen immediately at − 70 °C and stored until examination. Cryosections (6 μm thickness) of thrombi were attached to silane-coated slides. Sections were fixed in acetone at 4 °C for 10 min and air-dried for 5 min at room temperature. After further fixation in 4 w/v% paraformaldehyde (pH 7.0) at 4 °C for 10 min, the sections were washed in 10 mM Tris-HCl, pH 7.4, containing 150 mM NaCl (TBS) for 5 min, followed by incubation in 100 mM Na-phosphate 100 mM NaCl pH 7.5 buffer (PBS) containing 5 w/v% bovine serum albumin to eliminate nonspecific binding of antibodies. For double immunostaining, sections were first incubated overnight at 4 °C in 2 μg/mL mouse anti-human NE-digested FDP monoclonal antibody IF-123 (Mitsubishi Kagaku Iatron, Tokyo, Japan) [16] in TBS. Following washing with PBS, sections were treated with Alexa Fluor 555 (excitation 555 nm, emission 565 nm) carboxylic acid, succinimidyl ester conjugated donkey anti-mouse immunoglobulin antibody (Invitrogen, Oregon, USA) at 1:100 dilution to detect the IF-123. Subsequently slides were washed in PBS 3 times and blocked with 5 w/v% bovine serum albumin in PBS, then incubated with 2 μg/mL mouse anti-human fibrin monoclonal antibody ADI-350 (American Diagnostica, Pfungstadt, Germany) or mouse anti-human CD41 (platelet GPIIb/IIIa) antibody (Biodesign International, Saco, ME) for 30 min at 37 °C followed by detection of the second immune reaction with Alexa Fluor 488 (excitation 494 nm, emission 519 nm) 5-carboxamido-(propargyl), bis(triethylammonium salt)) *5-isomer* conjugated goat anti-mouse antibody (Invitrogen, Oregon, USA) at 1:100 dilution. After repeated washing with PBS, sections were mounted with 4’,6-diamidino-2-phenylindole (DAPI), which recognizes DNA, and images were taken with Eclipse E600 fluorescent microscope (Nikon, Japan) (Fig. 1).

### Image processing

Thrombus images acquired at 808 × 1104 pixel resolution were saved in JPEG format. All image pixels were allocated to the whole area of the thrombus section so that images did not display any useless information. Image processing was performed by self-devised Matlab scripts running in Matlab environment (Matlab 6.5.0 Release 13, The MathWorks, Inc., Natick, MA USA). RGB images have three channels (red, green, and blue) according to the three primary colours of light and triple system of notation was applied: the NE-degraded FDPs were allocated to the red channel, the fibrin or platelets were green and nuclei were blue in the same thrombus section. Thus, the separation of the thrombus constituents was based upon the image channels. After importing the thrombus files into the Matlab software environment the full colour RGB images were converted to double-precision images for processing. This process generates three individual monochromatic images representing the red, green, and blue components of the original image. This method enabled automatic and bias-free decomposition of the thrombus image and spatial localization of the thrombus constituents. The constituent intensities as a function of the space coordinates are illustrated on 3D plots in Fig. 1 C-E by color-coded intensity values. All statistical analyses were performed on integrated intensity values (region of interest was the whole image) or on intensity values normalized for leukocyte content (the integrated NE-FDP, fibrin and platelet signal of each thrombus image divided by the integrated nuclear-stain signal of the same image).

### Statistical analyses

Statistical calculations were implemented using Statistica 8.0 (StatSoft Inc., Tulsa, OK, USA). Linear regression model was applied for the quantification of the association between the neutrophil cell count and the NE-digested FDP content of the same thrombus sample on one side, and between the neutrophil cell count in the thrombus and in the blood samples of the same patient on the other. In order to identify similarity classes of patients based on the processes going on in the thrombi, we used hierarchical, agglomerative clustering analysis applying Ward's amalgamation rule and Euclidean distances [17]. This multivariate statistical procedure is an approach of exploratory data analysis, which aims at sorting different objects revealing meaningful groups in data. Classification puts “similar” thrombi into the same group (cluster), where similarity is defined by “distance” measures. In our analyses each thrombus (as an object to be classified) was described by the following sets of features: 1) a triplet of amount of elastase-digested fibrin, leukocyte content and platelet GPIIb/IIIa antigen; 2) a triplet of elastase-digested fibrin, leukocyte and fibrin content; 3) a duplet of normalized elastase-digested fibrin and normalized fibrin; 4) a duplet of normalized elastase-digested fibrin and normalized platelet antigen content. Distances are calculated between every pair of thrombi using these three or two “coordinates” of the thrombi. Based on the value of the distances, the applied agglomerative, hierarchical clustering builds groups by “amalgamation”, pairing the closest objects step-by-step into constantly growing clusters with increasing population number and re-calculating the distances between the newly formed larger groups until a hierarchical tree is formed. The output of the clustering is a tree diagram (or dendrogram) displaying the joining hierarchy, which allows the recognition of the main clusters. Cluster merge is marked by a horizontal line and each node in the graph represents a branched cluster. The vertical axis measures inter-cluster distance and each value on the axis refers to a criterion distance at which the respective clusters were joined into a new single cluster. Since statistical significance testing is not appropriate with this type of clustering, we used discriminant analysis for the verification of the identified clusters [17]. Discriminant function analysis tests if the clusters differ between each other significantly, i.e. if they are natural, distinct classes. All the clusters reported in the Results section proved to be significant with this statistical procedure. For the comparison of cluster mean and variance values Hotelling's multivariate T^2^ and F-statistics was implemented [17].

## Results

### Leukocytes in the structure of thrombi

**Fig. 2:**
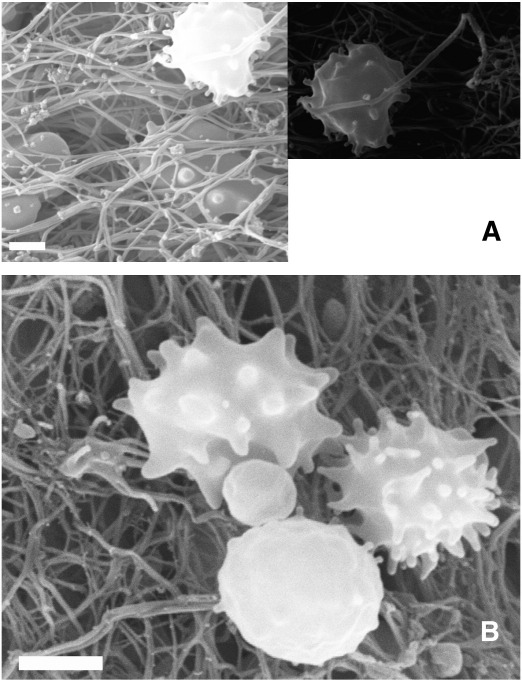
Leukocytes in the structure of thrombi. SEM images of a thrombus from pulmonary embolus (A) and implanted arterial graft (B) are shown. In panel A one of the leukocytes from the left image is focused in a separate frame to emphasize the tight proximity of the cell pseudopodia and the fibrin fiber. In panel B three leukocytes at different stages of pseudopodium formation surround a platelet. Scale bar = 2 μm.

SEM images of thrombi provided evidence of leukocytes forming pseudopodia in-between the fibrin fibers (Fig. 2A). Leukocytes could be observed in tight proximity with platelets and differences in pseudopodium formation indicated that they are in different stages of activation (Fig. 2B). Because SEM imaging allows only qualitative morphological evaluation of the interrelations between leukocytes, platelets and fibrin, the functional aspects of leukocyte presence with respect to thrombus dissolution were approached with quantitative data on neutrophil-related fibrin digestion.

### Correlation between elastase-digested fibrin and leukocyte content of thrombi

**Fig. 3:**
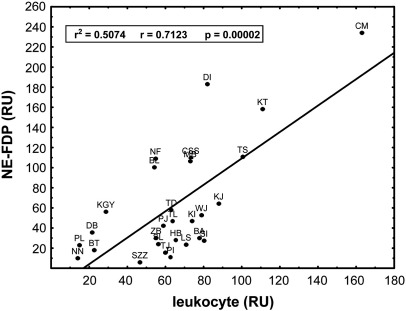
Correlation between elastase-digested fibrin and leukocyte content of thrombi. Fluorescence was assigned to NE-FDP or leukocytes according to the approach shown in Fig. 1. The association of the integrated intensity values (in relative units, RU) for the two constituents in the same image was evaluated by Pearson's correlation coefficient (r^2^ is the coefficient of determination). Different thrombi are indicated by patients’ initials.

Based on the fluorescent intensity values for NE-digested FDPs and nuclear DAPI staining of sections from surgically removed thrombi (Fig. 1), the association between the elastase-digested fibrin and the leukocyte content of the thrombi could be quantified by Pearson correlation coefficient (Fig. 3), which proved to be significant (r = 0.71, p = 0.00002). According to the coefficient of determination, 51 percent of the variability of the elastase digested fibrin content could be attributed to the presence of leukocytes. In general significant correlation does not necessarily imply causation, since correlation can also emerge from (hidden) common factors without direct causality. In this case, however, because neutrophils are the single known sources of NE and these cells represent 76 % of all leukocytes in thrombi [3], a causal relationship is highly probable. The remnant 49 percent variability, which cannot be explained by the quantity of leukocytes in thrombi, is probably related to factors determining the degree of activation and degranulation of leukocytes. In line with such a conclusion, no significant correlation was found, when a correlation coefficient between patients’ leukocyte count in blood and leukocyte content in thrombi was calculated (data not shown). This fact points to the importance of local processes involved in the formation of thrombi and cell-dependent thrombolysis.

### Classification of patients based on elastase-digested fibrin, leukocyte and platelet antigen content in thrombi

**Fig. 4:**
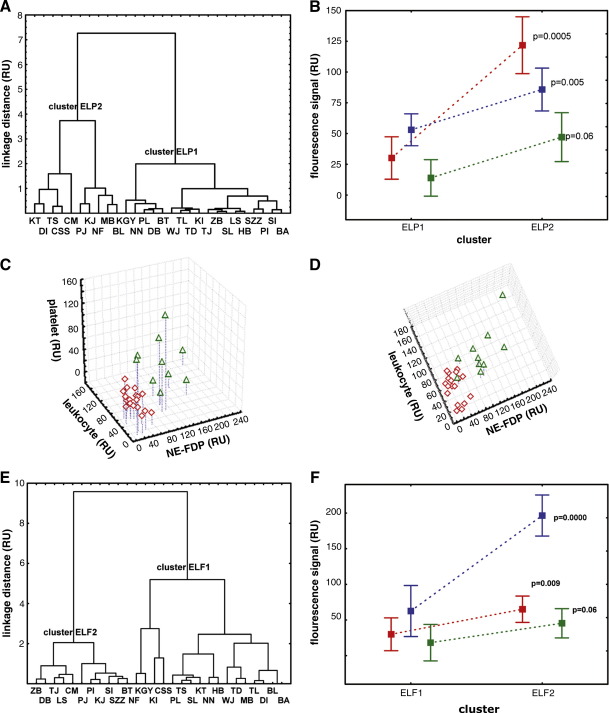
Clustering of patients according to the elastase-digested fibrin, leukocyte, platelet antigen and fibrin content of their thrombi. The thrombus constituents were quantified as illustrated in Fig. 1. Based on the NE-FDP, leukocyte and platelet antigen content two main clusters of patients (ELP1 and ELP2) were revealed by hierarchical, agglomerative clustering technique using Ward's method and Euclidean distances (A) and two main clusters (ELF1 and ELF2) were identified on the basis of NE-FDP, leukocyte and fibrin content (E). In panel B univariate comparison of the NE-FDP (red), leukocyte (blue) and platelet (green) content in clusters ELP1 and ELP2 is shown, whereas panel F presents the same analysis for clusters ELF1 and ELF2, where the green color stands for fibrin (mean and 95 % confidence intervals are indicated). Multiple correlation analysis was performed separately for the patients belonging to the clusters ELP1 shown with red symbols and ELP2 shown with green symbols (C, D).

Cluster analysis is an exploratory data analytic method, which does not require any *a priori* hypotheses about the phenomena under investigation, but rather lets the data speak. Thus, this analytic approach has definite advantages under the clinical conditions of our study, when little or no objective data are available about the age of thrombi. When the amount of elastase-digested fibrin, leukocyte content and platelet GPIIb/IIIa antigen were used for classification of thrombi, two main clusters (ELP1 and ELP2) emerged and these were verified by the discriminant function analysis. The size of cluster ELP1 is 18 and the cluster is more homogeneous than cluster ELP2, the size of which is 10 (Fig. 4A). We attempted to get a better insight into the clustering principles underlying the existence of these classes of thrombi by calculating multivariate means and variances of the clusters. Hotelling's multivariate statistics identified the clusters as significantly different in their thrombus composition (T^2^ = 96.0 F(3,24)=29.5, p < 0.00001). Thrombi isolated from patients of cluster ELP2 had significantly higher elastase-digested fibrin (p = 0.0005) content than thrombi of patients belonging to cluster ELP1 (Fig. 4B). The difference in platelet-related antigen content was nearly significant (p = 0.06). Since F-ratio test indicated inhomogeneity of variances in both cases, mean values were probed by Welch's method of separate variance estimate [17]. The mean values for leukocyte content (estimated from nuclear staining) were also different (p = 0.005), while variances did not differ significantly (Table 1). Moreover, we found that leukocyte content of thrombi predicted elastase-digested fibrin significantly in cluster ELP2 (multiple correlation R^2^ = 0.58), but not in cluster ELP1 (R^2^ = 0.25) (Fig. 4C-D). This association explains the greater variability of cluster ELP2, and that consequently it is less homogeneous than cluster ELP1. These results can be interpreted as a dynamical effect; inhomogeneity (greater variability) of cluster ELP2 might result from the patients being in a later phase of cell-related thrombolysis (due to variable invasion of leukocytes into the thrombus and their activation), while digestion has just begun in cluster ELP1.

### Classification of patients based on elastase-digested fibrin, leukocyte and fibrin content in thrombi

When classification was based on this second set of thrombus features, the same hierarchical, agglomerative clustering approach as above revealed two main clusters (ELF1 and ELF2) (Fig. 4E). The size of cluster ELF1 is 17, whereas that of ELF2 is 11. Hotelling's multivariate statistics again identified significant differences between the clusters based on their thrombus composition (T^2^ = 36.4 F(3,24)=11.2, p < 0.00009). The two clusters differed both in content of leukocytes (p = 0.009) and elastase-digested fibrin (p = 0.00000) according to univariate comparisons (Fig. 4F), while the amounts of fibrin were not significantly different (Table 2). Thrombi in cluster ELF1 contained lower numbers of leukocytes and therefore had lower amounts of elastase-digested fibrin. The variances of all constituents are significantly higher in cluster ELF2. Although these thrombi were sampled in a different session, the impression they produced about the dynamics of thrombolysis was similar to the one mentioned above in relation to ELP1 and ELP2.

### Evaluation of the thrombus constituents’ content (elastase-digested fibrin, platelet GPIIb/IIIa antigen and fibrin) as a normalized function of the leukocyte content

Because we found that thrombi showed significantly varying leukocyte content*,* potential interrelations of the other examined characteristics (amount of fibrin, NE-FDP and platelet in thrombi) can be evaluated on the basis of a parameter, which is independent of the leukocyte effects. To this end we applied normalization of the fluorescent signals by unit amount of leukocyte. Normalized values for NE-FDP, fibrin and platelet antigen were derived by dividing the integrated NE-FDP, fibrin and platelet signal of each thrombus image by their nuclear-stain signal.

**Fig. 5:**
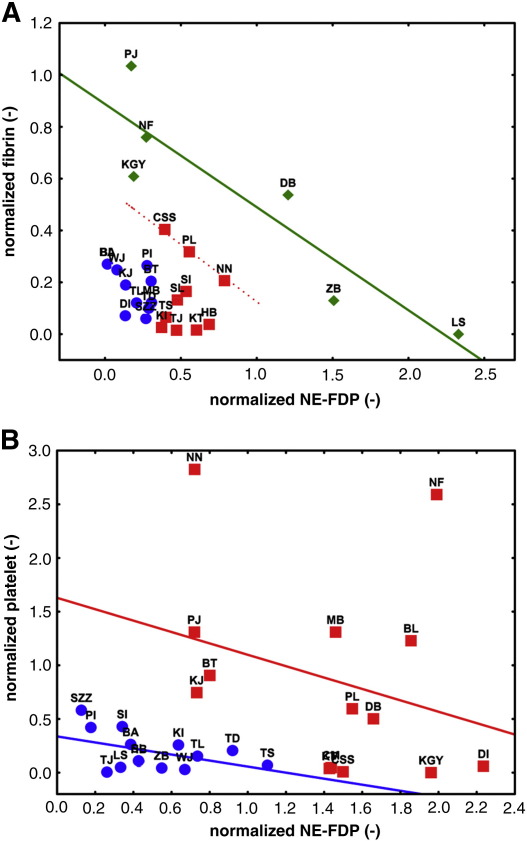
Elastase-digested fibrin, platelet GPIIb/IIIa antigen and fibrin content of thrombi normalized to their leukocyte content. The integrated fluorescence values for NE-FDP, platelet antigen and fibrin are divided by the integrated intensity for leukocytes in the same images. Cluster analysis was performed according to the combination of normalized NE-FDP and fibrin (A), which identified three classes of patients (REF1, blue; REF2, red; REF3, green), or the combination of normalized NE-FDP and platelet antigen (B), which indicates two classes of patients (REP1, blue; REP2, red). Continuous lines represent the linear regression analysis of all data in the cluster shown with the same color, whereas the dashed line indicates the regression to the data of only three patients (CSS, PL and NN).

Such an evaluation points to the role of additional local factors (e.g. temporal dynamics) superimposed on the cell-dependent thrombolysis. Clustering analysis was performed in two duplets of features: 1) normalized NE-FDP and normalized fibrin; 2) normalized platelet antigen and normalized NE-FDP. According to the first set of characteristics three clusters of patients emerged (Fig. 5A): REF1, REF2 and REF3. The thrombi in cluster REF1 contained small amounts of normalized NE-FDP and normalized fibrin. Cluster REF2 was characterized by higher amounts of normalized NE-FDP and even higher amounts of normalized fibrin. In cluster REF3 the normalized NE-FDP showed inverse ratio to the normalized fibrin. These characteristics of the REF1, REF2 and REF3 clusters can be interpreted in terms of the dynamics of thrombus development. At low fibrin content low cell-dependent thrombolytic activity is expressed (REF1). The process seems to start evolving in REF2 (as the amount of digested fibrin increases) especially in the case of patients CSS, PL and NN, whose thrombi contained both higher amounts of fibrin and digested fibrin. In these three patients an inverse relationship between the digested and undigested fibrin appeared according to linear regression with a negative slope of -0.3977. The linear regression of all data for cluster REF3 shows the same slope (Fig. 5A) and proved to be significant (p = 0.0025).

When normalized platelet antigen and normalized NE-FDP were used as characterizing variables in the cluster analysis, we gained insights into the role of platelets in cell-dependent fibrinolysis. Clustering classified the patients’ thrombi into two groups (Fig. 5B): REP1 and REP2. Thrombi of cluster REP1 were characterized by relatively small amounts of normalized NE-FDP and normalized platelet antigen was also low. In cluster REP2 normalized NE-FDP was in inverse ratio to normalized platelet antigen. It is plausible to assume that the cell-dependent fibrinolytic process was scarcely progressed in cluster REP1, but was very intense in cluster REP2. Thus, the degree of cell-dependent fibrinolysis is inversely proportional to the content of platelet antigen. The relationships between platelet and NE-FDP can also be described by linear regression in both REP1 and REP2 clusters, although the slopes (− 0.2813 and − 0.5307, respectively) do not differ significantly from zero.

## Discussion

Despite the early histological evidence for the role of neutrophilic leukocytes in the removal of fibrin [9,10], little is known about the quantitative significance of this alternative thrombolytic route *in vivo*. Using antibody raised against the NE-specific FDPs [16], three studies confirmed the elevation of these fibrinolytic products in the plasma of patients with disseminated intravascular coagulation and deep vein thrombosis, as well as after cardiopulmonary bypass surgery and major abdominal surgery [18–20], but no information is available on the local content of elastase-digested fibrin in human thrombi. The demand to acquire such information in humans is justified by the data from plasminogen knock-out mice [21], which show increased infiltration of thrombi by polymorphonuclear leukocytes, suggesting their compensatory role if the plasmin-dependent thrombolysis is deficient. Our study was undertaken in attempt to characterize in quantitative terms the local contribution of neutrophil elastase to the overall thrombolytic process in humans. Thrombi of twenty-eight patients subjected to thrombectomy were studied for the presence of NE-digested fibrin, platelet GPIIb/IIIa antigen, fibrin and nucleated cells. The single criterion for patient selection was the surgical indication for thrombectomy. Thus, the pathological background and localization of thrombi were heterogeneous, but most of the examined thrombi (22 out of 28) were atherosclerosis-related.

Simple visual inspection of the images of triple-stained thrombus sections produced the impression of a massive presence of NE-digested fibrin (Fig. 1A) and co-localization of nucleated cells and NE-FDP (Fig. 1B) in accordance with the visualization of activated leukocytes within the fibrin meshwork of thrombi (Fig. 2). However, objective analysis of the cumulative image data requires a quantitative and bias-free approach. Pixel decomposition of the truecolor images (Fig. 1C-E) provides a tool to allocate the intensity values of the separate color channels in space and thus the distribution of the thrombus constituents can be quantitatively characterized. This image processing technique allowed statistically sound conclusions from the global evaluation of the 28 patients’ thrombi. Thus, the visual impression of co-localization of NE-FDP and leukocytes was in line with the significant Pearson correlation coefficient between the elastase-digested fibrin and the leukocyte content of the thrombi (Fig. 3). The effect of the local accumulation of leukocytes in thrombi [3] seems to be causal with respect to the generation of NE-FDP. Our study is the first to verify quantitatively the *in vivo* association of the local presence of leukocytes and the cell-dependent degradation of fibrin. Because most of the thrombi evaluated in this study were of atherosclerotic origin, the cell-related thrombolysis may be a significant aspect of the resolution of atherothrombotic clots.

The platelet content of 10 mL blood is concentrated in a 400-μL volume of thrombi [22]. Consequently, it is essential to evaluate the thrombus platelet content in association with the local indicators of cell-dependent thrombolysis. In our analysis of thrombi with heterogeneous pathological origin and localization no *a priori* distinction criteria were used, all interrelations were established with bias-free statistical evaluation of the quantitative data from the complete set of available samples. When thrombi were classified according to the combination of platelet-, NE-FDP and leukocyte-related signals, two definite classes could be identified (Fig. 4A): a more homogeneous ELP1 cluster with lower NE-FDP, leukocyte and platelet content and a more heterogeneous ELP2 cluster with higher NE-FDP, leukocyte and platelet content. This refined classification shows that a subclass of patients (ELP1) could be discriminated, in which no correlation between leukocytes and NE-FDP exists, whereas in the more variable cluster (ELP2) a straightforward correlation of these two parameters could be established, which inevitably is stronger than the one in the general group of patients. A plausible explanation for the differences observed in the two classes of thrombi might be their age: the younger thrombi of ELP1 were scarcely affected by cell-dependent thrombolysis, whereas the aged ELP2 thrombi were in a more progressed stage of resolution. In the absence of direct measure of thrombus age, however, this interpretation remains hypothetic and its confirmation requires future studies. If cluster analysis is performed according to the combination of fibrin-, NE-FDP and leukocyte-related signals, two definite classes could be identified (Fig. 4E), but their constituents differ less than in the classification involving platelet content. This suggests a more significant role for platelets (than the total fibrin content) as a determinant of the cell-dependent thrombolysis, which is probably related to the localization of the thrombi examined in the present study (most of them are arterial, known to have higher platelet content than venous thrombi).

In order to eliminate the impact of the variable leukocyte content, the signals related to thrombus constituents (NE-FDP, fibrin, platelets) were normalized to the leukocyte signal and cluster analysis was performed on the basis of normalized NE-FDP and fibrin (yielding three classes: REF1, REF2 and REF3) or normalized NE-FDP and platelet values (yielding two classes: REP1 and REP2) (Fig. 5). Normalization by leukocyte content served to reveal the inverse relationship between the intact fibrin and NE-digested fibrin content, which did not emerge from the raw data. Thus, we found that the invasion of leukocytes in the thrombus could change its state as a function of the total fibrin content. Based on the characteristics of the REF1, REF2 and REF3 clusters, a dynamic picture of thrombus development emerges. At low fibrin content, low cell-dependent thrombolytic activity is expressed (REF1), which increases with the increase in fibrin (REF2) and reaches a dominant role, which curtails the fibrin content in the most advanced phase (REF3). It is worth noting that in spite of the fact that our analyses of thrombi represent just a momentary snapshot taken at the time of thrombectomy, they revealed several dynamic aspects and indirectly provided information on the temporal course of thrombus development and the relative prothrombotic and thrombolytic activity of the cells localized in the thrombi. The inverse relationship of NE-FDP and platelet antigen in the REP2 cluster points to the clot-stabilizing role of platelets against cell-dependent thrombolysis, which is in agreement with our earlier *in vitro* observations from NE-perfused platelet clots [23], whereas the association of low NE-FDP and low platelet content in REP1 is probably related to a low-level leukocyte stimulation by platelets [4] in this cluster.

In summary, quantitatively significant cell-dependent fibrinolytic activity in obliterative thrombi correlates with the invasion of leukocytes, the thrombolytic activity of which, in addition, shows dynamic interrelation with the thrombus fibrin and platelet content. Our structural data substantiate the need for further studies to clarify the causal interrelations between the fibrin and platelet content on one hand and the cell-dependent thrombolysis on the other.

## Disclosure of conflict of interests

None to declare.

## Figures and Tables

**Table 1:** Characteristics of patient clusters according to the content of elastase-digested fibrin, leukocytes and platelet antigen in thrombi. Sections of surgically removed thrombi were double- immunostained for NE-FDP and platelet GPIIb/IIIa antigen, leukocytes were identified by using nuclear staining. The thrombus constituents were quantified from the section images as described in Methods and the characteristics of the two patient clusters (ELP1 and ELP2) identified in Fig. 4A are presented.

Thrombus constituent	mean cluster ELP1	mean cluster ELP2	t	df	p	SD cluster ELP1	SD cluster ELP2	F-ratio variances	p variances
NE-FDP	30157	121835	− 5.04*	9.8	0.0005	16126.4	56218.2	12.2	0.000017
leukocyte	53069	85803	− 3.10	26	0.005	22798.7	33067.0	2.1	NS
platelet	13967	47095	− 2.10*	9.6	0.06	11933.7	49325.4	17.1	0.000002

* Student t-test with separate variance estimate.

**Table 2:** Characteristics of patient clusters according to the content of elastase-digested fibrin, leukocytes and fibrin in thrombi. Sections of surgically removed thrombi were double- immunostained for NE-FDP and fibrin antigen, leukocytes were identified by using nuclear staining. The thrombus constituents were quantified from the section images as described in Methods and the characteristics of the two patient clusters (ELF1 and ELF2) identified in Fig. 4E are presented.

Thrombus constituent	mean cluster ELF1	mean cluster ELF2	t	df	p	SD cluster ELF1	SD cluster ELF2	F-ratio variances	p variances
NE-FDP	29937	65072	2.83*	23.9	0.009	20223.7	44486.9	4.8	0.01
leukocyte	62553	196563	6.77*	25.4	0.0000	36721.8	67711.5	3.4	0.05
fibrin	18233	45381	1.94*	24.9	0.06	24594.0	48776.9	3.9	0.03

* Student t-test with separate variance estimate.
